# Racial Disparities in HIV Prevalence, Testing, and Care Among Men Who Have Sex with Men in Baltimore from 2008 to 2023: Fifteen Years of HIV Behavioral Surveillance

**DOI:** 10.1007/s10461-026-05031-7

**Published:** 2026-02-19

**Authors:** Danielle German, Molly Gribbin, Louis Spencer, James Burrell, Katrina Kennedy, Colin Flynn

**Affiliations:** 1 Department of Health, Behavior and Society, Johns Hopkins Bloomberg School of Public Health, Baltimore, MD USA; 2 Maryland Department of Health, Baltimore, MD USA

**Keywords:** HIV, Same gender loving/gay/bisexual, Men who have sex with men (MSM), Racial disparities, Baltimore

## Abstract

The Baltimore HIV epidemic among men who have sex with men (MSM) has been characterized by high HIV prevalence and stark racial disparities. We examined trends in HIV infection and service utilization among Baltimore MSM using data from CDC’s National HIV Behavioral Surveillance (NHBS) project from 2008 to 2023. A total of 2,180 venue-recruited MSM participated: 68% Black, 19% White, and 13% other/multiple racial identities. By 2023, HIV prevalence remained high (35.4% overall) and disproportionately concentrated among Black MSM (43.9%). From 2008 to 2023, predicted probability of HIV infection declined from 48% to 37% among Black MSM and remained stable at approximately 19% among White MSM. HIV service utilization exceeded 90% by 2023, particularly among Black MSM. These improvements reflect considerable community response and public health investment. Ending the HIV epidemic in Baltimore will require sustained prevention and treatment funding, continued efforts to reduce racial disparities, and focused attention on social determinants of health.

**Supplementary Information:**

The online version contains supplementary material available at https://doi.org/10.1007/s10461-026-05031-7.

## Resumen

La epidemia del VIH en Baltimore entre hombres que tienen sexo con hombres (HSH) se ha caracterizado por una alta prevalencia y marcadas disparidades raciales. Examinamos las tendencias en infección por VIH y utilización de servicios entre HSH de Baltimore utilizando datos del Sistema Nacional de Vigilancia del Comportamiento ante el VIH de los CDC de 2008 a 2023. Participaron un total de 2,180 HSH reclutados en lugares de encuentro: 68% negros, 19% blancos, 13% de otras o multiples identidades raciales. Para 2023, la prevalencia del VIH permanecía alta (35.4% en general) y se concentraba desproporcionadamente entre HSH negros (43.9%). De 2008 a 2023, la probabilidad predicha de infección por VIH disminuyó del 48% al 37% entre HSH negros y se mantuvo estable en aproximadamente el 19% entre los HSH blancos. La utilización de servicios de VIH superó el 90% en 2023, especialmente entre HSH negros. Estas mejoras reflejan una considerable respuesta comunitaria e inversión en salud pública. Acabar con la epidemia del VIH entre HSH en Baltimore requerirá financiamiento sostenido para la prevención y el tratamiento, esfuerzos continuos para reducir disparidades raciales y atención centrada en los determinantes sociales de la salud.

## Introduction

The Ending the HIV Epidemic (EHE) initiative called on U.S. jurisdictions to reduce HIV transmissions by at least 90% by 2030 [1, 2]. Achieving this ambitious goal requires a commitment to (1) ensuring the success of prevention strategies; (2) effective and accessible HIV treatment for those living with HIV/AIDS, and (3) strategies to address health disparities and the web of social and structural barriers that inhibit the implementation and sustainability of protective behaviors. At the five-year mark and at a time where continued investment in HIV prevention is uncertain, it is important to evaluate progress on key HIV-related metrics. This information can help to inform community priorities, recognize successes, and identify and address emergent concerns and other gaps in HIV prevention and response systems [3, 4].

Gay, bisexual, same gender loving, and other men who have sex with men (MSM) have been disproportionately affected by HIV since the earliest days of the U.S. epidemic due to a confluence of biological, social, and structural factors [5]. After a period of decline in new infections attributed to community response and noted successes in HIV prevention and treatment [1–3], newly diagnosed HIV among MSM began to climb again in the early 1990’s [4]. This resurgence highlighted gaps in the HIV response and stark racial disparities in the MSM HIV epidemic [6, 7]. The trajectory of racial disparities within the MSM HIV epidemic in the U.S. has been noted as a public health and social justice failure anchored in systems of social and structural stigma that limited attention to the experiences, needs, and strengths of Black and African-American MSM within the U.S. response to HIV [8, 9].

Currently, MSM have a one in six lifetime risk of HIV infection compared to one in 524 among heterosexual men and approximately one-quarter of MSM are estimated to have been diagnosed with HIV [10]. Approximately two-thirds of new infections are among MSM, and Black and African-American men continue to be disproportionately represented among new infections [11]. HIV prevalence among Black and African-American MSM nationwide is estimated to be 48% compared to 21% among white MSM [10]. Ongoing monitoring of epidemiological and behavioral trends and potential disparities on a local level is critical to evaluate progress towards achieving EHE goals and inform continued programmatic priorities.

Baltimore has historically been among the U.S. cities with the highest numbers of HIV cases and the highest prevalence of HIV among MSM in the U.S. Furthermore, the HIV epidemic among MSM in this city has been heavily concentrated among Black and African American MSM, who currently account for 80% of prevalent infections among MSM [12]. Studies have consistently demonstrated severe racial disparities in HIV infection among Baltimore MSM with Black and African American men between 2.5 and 4 times as likely to test HIV positive compared to white MSM [13], rates as high as 1 in 2 among Black and African American men [14], and elevated prevalence relative to local and national comparison cities [14, 15].

Overall, the HIV epidemic in Baltimore continues to be concentrated among non-Hispanic Black and African American MSM but there are indications of substantial improvement [12]. A total of 104 new HIV infections diagnosed among men in 2023 were attributed to male-to-male sexual transmission, reducing by more than half the number diagnosed annually from 2004 to 2012 [12]. There has been a steady decline in the number of new infections among MSM since 2010, when the increasing trend of new infections that began in the early 1990s reversed course. However, these data provide information only about MSM who have been diagnosed with HIV and analyses of trends tend to be limited by the small number of demographic characteristics that are systematically collected for surveillance purposes.

HIV behavioral surveillance was established by CDC in 2004 [16] to monitor trends and patterns in HIV infection, service utilization, and related social and behavioral factors over time among populations highly affected by HIV, including MSM, in U.S. jurisdictions with high HIV case numbers, including Baltimore. Thus, HIV behavioral surveillance data are a key source of information to examine key HIV indicators over time and evaluate the persistence of racial disparities.

We used Baltimore National HIV Behavioral Surveillance [17] (NHBS; locally known as the BEhavioral SURveillance REsearch study, or BESURE) data collected among MSM in 2008, 2011, 2014, 2017, and 2023 to examine patterns in HIV prevalence, diagnosis, testing, and care by race over time among MSM in the Baltimore metro region.

## Methods

### Sample

NHBS is designed to monitor prevalence and trends in HIV, related behaviors, and service utilization among key populations in U.S. jurisdictions highly affected by HIV. In Baltimore NHBS is implemented as a partnership between the Johns Hopkins School of Public Health and the Maryland Department of Health. Eligibility criteria include being 18 years of age or older, a Baltimore metropolitan statistical area [18] resident, and English or Spanish speaking. Eligibility for MSM cycles additionally includes being male and reporting sex with another man in the past year. This study uses Baltimore NHBS data from eligible MSM 2008, 2011, 2014, 2017, 2023 (MSM2-MSM7) (*n* = 501, 452, 502, 415, 310, respectively).

### Sample Recruitment and Data Collection

BESURE MSM cycles used venue-based time-location sampling following the standardized NHBS protocol [16]. Extensive formative research among MSM and key stakeholders identified venues, time periods, and operational considerations for participant recruitment. Venues included primarily parks, bars, restaurants, events and street locations that were frequently used by MSM. Eligible venues and venue-specific day-time periods that met MSM attendance, logistical, and safety criteria composed a monthly sampling frame. Participant recruitment took place at randomly sampled venue day-time events, where men who entered venues were counted and systematically approached for screening, consent, and study participation.

Eligible participants who provided informed consent completed a socio-behavioral survey and optional HIV pre-and-post-test counseling in a secure and private mobile van, a community-based research site, or other private space. HIV testing was conducted using HIV rapid tests and confirmatory laboratory-based testing on collected blood specimens.

The NHBS-Baltimore protocol and all local materials were approved by Institutional Review Boards at the Johns Hopkins Bloomberg School of Public Health and Maryland Department of Health.

### Measures

We considered a set of outcome variables associated with the HIV cascade [19]: HIV positive status, new HIV diagnoses, HIV testing, HIV care, and use of anti-retroviral therapy (ART). All participants were asked if they have ever had an HIV test; and then if they have ever tested positive for HIV. Those with a prior HIV test who never tested positive for HIV were then asked the month and year of their most recent HIV test the results of their most recent test if received. Those who reported any prior positive HIV test result were asked if they have ever been seen by a doctor, nurse, or other health care provider for a medical evaluation or care related to HIV infection, recency of last visit, and whether they are currently taking antiretroviral medications to treat HIV infection. HIV status was based on the results of laboratory HIV tests and new HIV diagnoses were determined based on the combined results of laboratory testing and self-reported HIV status (e.g., positive HIV test and no reported prior HIV positive diagnosis).

To evaluate the impact of time (e.g., year of data collection), we assessed each of these outcomes in the presence of key socio-demographic covariates commonly associated with HIV outcomes. These included age, race/ethnicity (Hispanic, yes/no; then racial group: Black or African-American; white; and American Indian or Alaska native, Asian, Native Hawaiian or Other Pacific Islander grouped with multiple race and Hispanic ethnicity for analysis given small samples), sexual orientation (gay/lesbian/homosexual compared to bisexual, heterosexual, other), injection drug use ever, homelessness in the past 12 months (yes/no), and type of recruitment venue.

### Statistical Analysis

We examined prevalence of and potential socio-demographic disparities for each outcome variable using adjusted logistic models calculated across time for the total sample and separately by race. We then compared marginal probabilities estimated by standardizing the sample to different waves to evaluate the effect of time. Using parameter estimates from the corresponding logistic models we obtained predicted probabilities of each outcome of interest for each person in each NHBS cycle. We compared distributions of these predicted probabilities across cycles, accounting for the demographic characteristics of each cycle, using the Kruskal-Wallis test – a method well-suited for multiple group comparisons. We then applied the Dwass, Steel, Critchlow-Fligner method to identify which specific pairs of cycles differed from each other while accounting for the increased risk of error associated with multiple comparisons. The estimation was done with SAS software using proc npar1way procedure. We conducted further analyses stratified by race, given the stark and persistent racial disparities in HIV among Baltimore MSM.

## Results

Table 1 shows the descriptive characteristics of the overall sample for each wave. Analyses were standardized in comparison to characteristics of the 2008 sample (*n* = 501). In this wave, approximately half of participants were under 30, close to 40% had annual income under $20,000, almost half had high school education or less, and approximately two-thirds identified as gay. 6% reported a history of injection use, 16% reported past year unhoused status, and approximately 80% were recruited from a bar or club setting. Less than 10% reported prior study participation based on recognition of study name, logo, van, or office location.

**Table 1:** Demographic characteristics of eligible Baltimore NHBS-MSM respondents by wave, 2008–2023 (Total *n* = 2180)

	2008 (*N* = 501)	2011 (*N* = 452)	2014 (*N* = 502)	2017 (*N* = 415)	2023 (*n* = 310)	Total (*N* = 2180)
Variables	n (%)	n (%)	n (%)	n (%)	n (%)	n (%)
Age						
< 30	249 (49.7%)	226 (50.0)	229 (45.6)	149 (35.9)	60 (19.4)	913 (41.9)
30+	252 (50.3)	226 (50.0)	273 (54.4)	266 (64.1)	250 (80.7)	1267 (58.1)
Race						
Black	323 (64.5)	348 (77.0)	326 (64.9)	281 (67.7)	212 (68.4)	1490 (68.4)
White	116 (23.2)	64 (14.2)	111 (22.1)	65 (15.7)	54 (17.4)	410 (18.8)
Another/Multiple	62 (12.4)	40 (8.9)	65 (13.0)	69 (16.6)	44 (14.2)	280 (12.8)
Income						
<$20,000	190 (38.2)	236 (54.9)	201 (42.3)	183 (44.6)	127 (41.2)	937 (44.2)
$20,000–49,999	190 (38.2)	122 (28.4)	152 (32.0)	138 (33.7)	73 (23.7)	675 (31.8)
$50,000+	118 (23.7)	72 (16.7)	122 (25.7)	89 (21.7)	108 (35.1)	509 (24.0)
Education						
HS/GED or less	235 (46.9)	250 (55.4)	199 (39.6)	175 (42.2)	113 (36.5)	972 (44.6)
Some College	170 (33.9)	141 (31.3)	145 (28.9)	132 (31.8)	90 (29.0)	678 (31.1)
BA+	96 (19.2)	60 (13.3)	158 (31.5)	108 (26.0)	107 (34.5)	529 (24.3)
Sexual Orientation						
Gay	345 (68.9)	292 (64.9)	349 (69.5)	272 (65.9)	186 (61.0)	1444 (66.5)
Bisexual	148 (29.5)	146 (32.4)	138 (27.5)	128 (31.0)	113 (37.1)	673 (31.0)
Heterosexual	8 (1.6)	12 (2.7)	15 (3.0)	13 (3.2)	6 (2.0)	54 (2.5)
Homelessness						
Never or not in past year	421 (84.0)	377 (84.0)	446 (88.8)	355 (85.5)	257 (82.9)	1856 (85.3)
Past year	80 (16.0)	72 (16.0)	56 (11.2)	60 (14.5)	53 (17.1)	321 (14.8)
Injection drug use ever						
No	473 (94.4)	410 (91.3)	466 (92.8)	379 (91.3)	276 (89.0)	2004 (92.1)
Yes	28 (5.6)	39 (8.7)	36 (7.2)	36 (8.7)	34 (11.0)	173 (8.0)
Venue type						
Bar/Club	390 (80.4)	314 (69.5)	347 (69.1)	259 (62.4)	154 (49.7)	1464 (67.7)
Sex Environment/ Street Location/Park	61 (12.6)	53 (11.7)	16 (3.2)	26 (6.3)	83 (26.8)	239 (11.0)
Other	34 (7.0)	85 (18.8)	139 (27.7)	130 (31.3)	73 (23.6)	461 (21.3)
Reported previous BESURE participation	-	21 (4.7%)	57 (12.2%)	54 (14.2%)	39 (12.7%)	172 (8.2%)

### Prevalence of HIV Outcomes Over Time

#### HIV Prevalence and New Diagnoses

Table 2 shows notable changes in HIV outcomes over time and differences by race/ethnicity. Overall HIV prevalence was 39% in 2008, with a small decrease to 35% in 2023 and some slight variation over time. The highest HIV prevalence was among Black MSM at all time points (46% in 2008 and 44% in 2023) and racial disparities in HIV prevalence remained relatively stable: 2.5 times higher among Black MSM compared to white MSM in 2008 and 2.8 times higher in 2023. The proportion of total MSM with new HIV diagnoses decreased substantially in all groups (from 28% in 2008 to 7% in 2023), with the most notable decreases observed among Black MSM (34% in 2008 to 9% in 2023) and a reduction to zero among white MSM as of 2017.

**Table 2:** HIV prevalence and service utilization indicators among eligible Baltimore NHBS-MSM respondents by race and by wave, 2008–2023 (Total *n* = 2180)

	2008 *n* (%)	2011 *n* (%)	2014 *n* (%)	2017 *n* (%)	2023 *n* (%)
Total MSM	*n* = 501	*n* = 452	*n* = 502	*n* = 415	*n* = 310
HIV positive test result ^1^	173 (38.7)	171 (42.4)	139 (30.6)	138 (36.9)	98 (35.4)
Newly diagnosed with HIV ^1^	123 (27.8)	116 (28.9)	55 (12.1)	28 (7.5)	20 (7.3)
Ever tested for HIV	456 (91.2)	409 (90.5)	474 (94.4)	400 (96.6)	302 (97.4)
Tested in past year	270 (54.4)	252 (56.0)	280 (61.0)	222 (54.6)	151 (49.4)
HIV care visit in past year ^2^	43 (87.8)	45 (80.4)	77 (87.5)	109 (96.5)	78 (92.9)
Taking ARV ^2^	27 (55.1)	40 (70.2)	77 (87.5)	104 (91.2)	80 (94.1)
Undetectable viral load ^3^	-	-	-	97 (92.4)	71 (97.3)
Black	*n* = 323	*n* = 348	*n* = 326	*n* = 281	*n* = 212
HIV positive test result ^1^	132 (46.2)	148 (47.9)	120 (41.2)	113 (43.8)	82 (43.9)
Newly diagnosed with HIV ^1^	95 (33.5)	105 (34.1)	49 (16.8)	24 (9.3)	17 (9.1)
Ever tested for HIV	294 (91.0)	317 (91.1)	312 (95.7)	275 (98.2)	208 (98.1)
Tested in past year	180 (55.9)	197 (56.8)	180 (61.9)	143 (52.2)	98 (46.9)
HIV care visit in past year ^2^	30 (85.7)	36 (81.8)	63 (86.3)	88 (96.7)	66 (93.0)
Taking ARV ^2^	17 (48.6)	31 (68.9)	64 (87.7)	85 (92.4)	68 (94.4)
Undetectable viral load ^3^	-	-	-	78 (91.8)	60 (98.4)
White	*n* = 116	*n* = 64	*n* = 111	*n* = 65	*n* = 54
HIV positive test result ^1^	19 (18.3)	9 (15.5)	9 (8.7)	10 (18.2)	8 (15.7)
Newly diagnosed with HIV ^1^	7 (6.9)	5 (8.6)	2 (1.9)	0 (0)	0 (0)
Ever tested for HIV	108 (93.9)	54 (84.4)	100 (90.1)	59 (90.8)	52 (96.3)
Tested in past year	53 (46.9)	32 (50.8)	64 (59.8)	33 (50.8)	28 (51.9)
HIV care visit in past year ^2^	12 (100)	2 (50.0)	8 (100.0)	11 (100.0)	7 (87.5)
Taking ARV ^2^	10 (83.3)	4 (100.0)	8 (100.0)	9 (81.8)	7 (87.5)
Undetectable viral load ^3^	-	-	-	10 (90.9)	6 (85.7)
Another/Multiple race	*n* = 62	*n* = 40	*n* = 65	*n* = 69	*n* = 44
HIV positive test result ^1^	22 (38.6)	14 (38.9)	10 (16.7)	15 (24.6)	8 (20.5)
Newly diagnosed with HIV ^1^	21 (36.8)	6 (16.7)	4 (6.7)	4 (6.6)	3 (7.7)
Ever tested for HIV	54 (87.1)	38 (95.0)	62 (95.4)	66 (95.7)	42 (95.5)
Tested in past year	37 (60.7)	23 (57.5)	36 (59.0)	46 (67.7)	25 (58.1)
HIV care visit in past year ^2^	1 (50.0)	7 (87.5)	6 (85.7)	10 (90.9)	5 (100.0)
Taking ARV ^2^	0 (0)	5 (62.5)	5 (71.4)	10 (90.9)	5 (100)
Undetectable viral load ^3^	-	-	-	9 (100.0)	5 (100)

Denominators may vary due to missing data

^1^ Among those with valid HIV test result

^2^ Among those with self-reported HIV diagnosis

^3^ Among those with self-reported HIV viral load test, ever

#### Lifetime and Past Year HIV Testing

Overall prevalence of HIV testing increased from 91% in 2008 to 97% in 2023. Among those not previously diagnosed, HIV testing in the past year changed from 54% to 49%. HIV testing trends among Black MSM were mixed: while lifetime testing increased substantially from 91% in 2008 to 98% in 2023, annual testing among those without a previous HIV diagnosis decreased from 56% to 47% during the same period, following an initial improvement between 2008 and 2014. Lifetime testing also increased among white and MSM of another or multiple racial and ethnic identities, but past year testing only increased between 2008 and 2023 among white MSM (47% to 52%).

#### HIV Care, Treatment, and Viral Suppression

HIV care utilization improved slightly overall but varied by race. Among those with a previous HIV diagnosis, past year HIV care visits increased from 88% in 2008 to 93% in 2023. This improvement was primarily among Black MSM (from 86% to 93%), whereas there was a decline among white MSM (from 100% to 88%). ART use almost doubled between 2008 (55%) and 2023 (94%), but this change was primarily among Black MSM (from 49% in 2008 to 94% in 2023) and relatively small among white MSM (from 83% in 2008 to 87% in 2023). Viral suppression among those with a previous HIV diagnosis and care visit was measured only in 2017 and 2023. During this period, proportion suppressed increased from 92% to 97% in the full sample. However, the improvement was only among Black MSM (92% to 98%). Viral suppression remained stable at 100% among MSM of another or multiple racial and ethnic identities and decreased slightly among MSM who were white (91% to 86%).

### Sociodemographic Correlates of HIV Outcomes Over Time

Table 3 shows results of multivariate logistic models of sociodemographic characteristics on HIV outcomes for each wave. Odds of HIV positive status were substantially and consistently higher among MSM who were over 30 and those who were Black. Those who identified as gay had two to three times higher odds of being HIV positive in most waves and there was a significant association between homelessness and HIV diagnosis in 2008, but neither association was significant by 2023. Odds of newly diagnosed HIV infection were substantially lower among white MSM from 2008 to 2014 and among MSM who identified as bisexual, heterosexual, or other sexual orientation in 2008 and 2011, but these associations were not significant in later waves and no other variables were significantly associated.

**Table 3:** Multivariate associations of sociodemographic characteristics with HIV outcomes by wave among eligible Baltimore NHBS-MSM participants, 2008–2023

			2008 *N* = 501	2011 *N* = 452	2014 *N* = 502	2017 *N* = 415	2023 *N* = 310	
			AOR (95% CI)	AOR (95% CI)	AOR (95% CI)	AOR (95% CI)	AOR (95% CI)	
HIV positive test result			*n* = 432	*n* = 398	*n* = 455	*n* = 372	*n* = 272	
Age 30 or older			**1.90 (1.23**,** 2.92)**	**2.46 (1.51**,** 4.01)**	**2.12 (1.34**,** 3.36)**	**1.65 (1.01**,** 2.70)**	**5.14 (2.04**,** 12.96)**	
Race		White	1.00 (--)	1.00 (--)	1.00 (--)	1.00 (--)	1.00 (--)	
Black			**5.03 (2.74**,** 9.25)**	**8.37 (3.72**,** 18.84)**	**9.20 (4.34**,** 19.49)**	**4.20 (1.95**,** 9.05)**	**4.61 (1.99**,** 10.69)**	
Another/Multiple			**3.79 (1.73**,** 8.28)**	**4.64 (1.61**,** 13.34)**	**2.45 (0.92**,** 6.55)**	1.42 (0.55, 3.67)	1.48 (0.48, 4.56)	
Ever injected drugs			1.30 (0.49, 3.46)	1.47 (0.64, 3.39)	2.26 (0.98, 5.23)	0.93 (0.39, 2.25)	0.81 (0.31, 2.07)	
Sexual orientation Gay			1.00 (--)	1.00 (--)	1.00 (--)	1.00 (--)	1.00 (--)	
Bisexual			**0.54 (0.34**,** 0.87)**	**0.26 (0.15**,** 0.44)**	**0.58 (0.35**,** 0.97)**	**0.28 (0.16**,** 0.49)**	0.57 (0.31, 1.03)	
Heterosexual			< 0.001 (< 0.001, > 999.99)	**0.06 (0.01**,** 0.54)**	0.22 (0.04, 1.15)	**0.08 (0.01**,** 0.72)**	0.67 (0.10, 4.57)	
Homeless in past year			**1.84 (1.06**,** 3.20)**	0.86 (0.47, 1.57)	1.46 (0.73, 2.90)	1.12 (0.57, 2.19)	1.24 (0.56, 2.74)	
Venue type		Bar/Club	1.00 (--)	1.00 (--)	1.00 (--)	1.00 (--)	1.00 (--)	
Sex Environment/Street Location/Park			0.82 (0.45, 1.50)	1.55 (0.76, 3.15)	2.44 (0.76, 7.81)	1.58 (0.62, 4.01)	0.96 (0.50, 1.84)	
Other			0.89 (0.40, 2.00)	1.25 (0.70, 2.25)	0.92 (0.56, 1.51)	1.51 (0.92, 2.47)	0.82 (0.42, 1.62)	
Newly diagnosed with HIV (among total sample)			*n* = 427	*n* = 397	*n* = 455	*n* = 372	*n* = 270	
Age 30 or older			1.36 (0.86, 2.17)	1.62 (0.98, 2.66)	**2.12 (1.10**,** 4.09)**	0.68 (0.30, 1.57)	> 999.99 (< 0.001, > 999.99)	
Race	White		1.00 (--)	1.00 (--)	1.00 (--)	1.00 (--)	1.00 (--)	
Black			**8.11 (3.47**,** 18.98)**	**6.24 (2.36**,** 16.54)**	**11.20 (2.62**,** 47.92)**	> 999.99 (< 0.001, > 999.99)	> 999.99 (< 0.001, > 999.99)	
Another/Multiple			9.56 (3.59, 25.51)	2.11 (0.57, 7.73)	4.07 (0.71, 23.30)	> 999.99 (< 0.001, > 999.99)	> 999.99 (< 0.001, > 999.99)	
Ever injected drugs			1.68 (0.59, 4.82)	1.61 (0.71, 3.65)	2.18 (0.84, 5.67)	1.19 (0.25, 5.79)	0.87 (0.17, 4.47)	
Sexual orientation Gay			1.00 (--)	1.00 (--)	1.00 (--)	1.00 (--)	1.00 (--)	
Bisexual			0.65 (0.39, 1.06)	**0.59 (0.35**,** 0.99)**	1.27 (0.67, 2.41)	0.82 (0.33, 2.01)	1.41 (0.52, 3.86)	
Heterosexual			< 0.001 (< 0.001, > 999.99)	< 0.001 (< 0.001, > 999.99)	0.49 (0.06, 4.37)	< 0.001 (< 0.001, > 999.99)	< 0.001 (< 0.001, > 999.99)	
Homeless in past year			1.57 (0.89, 2.76)	0.85 (0.45, 1.63)	1.47 (0.65, 3.34)	1.72 (0.63, 4.73)	1.04 (0.28, 3.82)	
Venue type	Bar/Club		1.00 (--)	1.00 (--)	1.00 (--)	1.00 (--)		
Sex Environment/Street Location/Park			1.08 (0.58, 1.98)	1.93 (0.95, 3.94)	1.44 (0.36, 5.71)	< 0.001 (< 0.001, > 999.999)	2.64 (0.91, 7.67)	
Other			0.83 (0.34, 2.03)	0.80 (0.43, 1.50)	1.12 (0.58, 2.15)	1.27 (0.56, 2.89)	0.80 (0.19, 3.30)	
Ever tested for HIV			*n* = 484	*n* = 446	*n* = 502	*n* = 412	*n* = 305	
Age 30 or older			0.67 (0.35, 1.30)	0.51 (0.25, 1.05)	1.21 (0.54, 2.74)	2.74 (0.86, 8.75)	9.55 (1.94, 47.09)	
Race		White	1.00 (--)	1.00 (--)	1.00 (--)	1.00 (--)	1.00 (--)	
Black			0.67 (0.27, 1.67)	**2.33 (1.01**,** 5.37)**	**2.88 (1.19**,** 6.97)**	**8.00 (2.05**,** 31.25)**	6.30 (0.75, 52.82)	
Another/Multiple			0.41 (0.13, 1.25)	4.31 (0.84, 22.12)	2.46 (0.65, 9.33)	3.04 (0.68, 13.73)	1.98 (0.21, 18.59)	
Ever injected drugs			1.39 (0.29, 6.63)	1.90 (0.53, 6.87)	0.61 (0.15, 2.44)	1.22 (0.13, 11.10)	> 999.99 (< 0.001, > 999.99)	
Sexual orientation Gay			1.00 (--)	1.00 (--)	1.00 (--)	1.00 (--)	1.00 (--)	
Bisexual			0.94 (0.46, 1.90)	0.53 (0.26, 1.09)	1.12 (0.42, 2.97)	0.49 (0.15, 1.64)	0.35 (0.07, 1.66)	
Heterosexual			> 999.99 (< 0.001, > 999.99)	0.46 (0.09, 2.42)	1.16 (0.13, 10.61)	> 999.99 (< 0.001, > 999.99)	> 999.99 (< 0.001, > 999.99)	
Homeless in past year			0.61 (0.28, 1.35)	0.85 (0.36, 2.03)	0.71 (0.22, 2.36)	0.43 (0.10, 1.93)	0.26 (0.04, 1.75)	
Venue type		Bar/Club	1.00 (--)	1.00 (--)	1.00 (--)	1.00 (--)	1.00 (--)	
Sex Environment/Street Location/Park			1.39 (0.51, 3.82)	**0.34 (0.14**,** 0.82)**	**0.16 (0.04**,** 0.66)**	0.64 (0.07, 5.70)	0.33 (0.05, 2.20)	
Other			1.79 (0.40, 8.02)	0.90 (0.38, 2.13)	1.09 (0.44, 2.69)	1.15 (0.29, 4.52)	0.21 (0.03, 1.48)	
HIV test in past year			*n* = 440	*n* = 397	*n* = 414	*n* = 302	*n* = 218	
Age 30 or older			**0.58 (0.39**,** 0.87)**	**0.35 (0.22**,** 0.55)**	**0.41 (0.26**,** 0.64)**	0.66 (0.37, 1.17)	1.12 (0.55, 2.27)	
Race	White		1.00 (--)	1.00 (--)	1.00 (--)	1.00 (--)	1.00 (--)	
Black			1.44 (0.86, 2.42)	1.29 (0.71, 2.35)	1.37 (0.82, 2.28)	**2.25 (1.16**,** 4.39)**	1.67 (0.80, 3.48)	
Another/Multiple			1.25 (0.62, 2.52)	1.82 (0.71, 4.69)	0.99 (0.49, 1.97)	**2.52 (1.06**,** 5.97)**	1.41 (0.56, 3.56)	
Ever injected drugs			0.81 (0.33, 1.99)	1.19 (0.55, 2.57)	0.75 (0.33, 1.72)	0.82 (0.33, 2.08)	1.32 (0.47, 3.73)	
Sexual orientation Gay			1.00 (--)	1.00 (--)	1.00 (--)	1.00 (--)	1.00 (--)	
Bisexual			**0.51 (0.32**,** 0.80)**	0.84 (0.52, 1.35)	1.34 (0.79, 2.25)	**0.44 (0.24**,** 0.78)**	0.66 (0.35, 1.22)	
Heterosexual			0.66 (0.16, 2.82)	0.94 (0.25, 3.55)	3.99 (0.82, 19.38)	0.61 (0.16, 2.40)	0.87 (0.07, 10.93)	
Homeless in past year			0.82 (0.47, 1.44)	1.25 (0.68, 2.28)	0.81 (0.40, 1.65)	1.47 (0.68, 3.21)	0.44 (0.19, 1.03)	
Venue type	Bar/Club		1.00 (--)	1.00 (--)	1.00 (--)	1.00 (--)	1.00 (--)	
Sex Environment/Street Location/Park			1.21 (0.65, 2.24)	0.75 (0.39, 1.45)	0.47 (0.13, 1.69)	1.05 (0.33, 3.29)	1.05 (0.53, 2.07)	
Other			1.24 (0.55, 2.80)	0.64 (0.37, 1.14)	0.86 (0.54, 1.38)	1.16 (0.63, 2.12)	1.36 (0.63, 2.96)	
HIV care visit in past year			*n* = 47	*n* = 55	*n* = 88	*n* = 113	*n* = 83	
Age 30 or older			> 999.999 (< 0.001, > 999.999)	0.59 (0.10, 3.31)	2.48 (0.56, 11.02)	2.11 (0.24, 18.22)	1.02 (0.07, 15.68)	
Race		White	1.00 (--)	1.00 (--)	1.00 (--)	1.00 (--)	1.00 (--)	
Black			< 0.001 (< 0.001, > 999.999)	2.77 (0.29, 26.59)	< 0.001 (< 0.001, > 999.999)	< 0.001 (< 0.001, > 999.999)	4.59 (0.31, 68.13)	
Another/Multiple			0.21 (< 0.001, > 999.999)	4.72 (0.21, 108.86)	< 0.001 (< 0.001, > 999.999)	< 0.001 (< 0.001, > 999.999)	> 999.999 (< 0.001, > 999.999)	
Ever injected drugs			> 999.999 (< 0.001, > 999.999)	> 999.99 (< 0.001, > 999.999)	> 999.999 (< 0.001, > 999.999)	> 999.999 (< 0.001, > 999.999)	> 999.999 (< 0.001, > 999.999)	
Sexual orientation Gay			1.00 (--)	1.00 (--)	1.00 (--)	1.00 (--)	1.00 (--)	
Bisexual			< 0.001 (< 0.001, > 999.999)	0.95 (0.08, 11.90)	0.56 (0.09, 3.60)	> 999.999 (< 0.001, > 999.999)	0.16 (0.02, 1.12)	
Heterosexual			-	< 0.001 (< 0.001, > 999.999)	< 0.001 (< 0.001, > 999.999)	> 999.999 (< 0.001, > 999.999)	21.16 (< 0.001, > 999.999)	
Homeless in past year			< 0.001 (< 0.001, > 999.999)	1.52 (0.11, 20.13)	> 999.999 (< 0.001, > 999.999)	0.51 (0.04, 7.12)	0.34 (0.02, 6.02)	
Venue type		Bar/Club	1.00 (--)	1.00 (--)	1.00 (--)	1.00 (--)	1.00 (--)	
Sex Environment/Street Location/Park			> 999.999 (< 0.001, > 999.999)	> 999.99 (< 0.001, > 999.999)	> 999.999 (< 0.001, > 999.999)	< 0.001 (< 0.001, > 999.999)	> 999.999 (< 0.001, > 999.999)	
Other			> 999.999 (< 0.001, > 999.999)	0.72 (0.13, 4.04)	**0.13 (0.03**,** 0.63)**	< 0.001 (< 0.001, > 999.999)	0.64 (0.09, 4.56)	
Taking ARV			*n* = 47	*n* = 56	*n* = 88	*n* = 114	*n* = 84	
Age 30 or older			**20.96 (3.03**,** 144.92)**	1.26 (0.28, 5.61)	1.38 (0.34, 5.58)	1.75 (0.36, 8.56)	< 0.001 (< 0.001, > 999.999)	
Race		White	1.00 (--)	1.00 (--)	1.00 (--)	1.00 (--)	1.00 (--)	
Black			0.13 (0.01, 1.88)	< 0.001 (< 0.001, > 999.999)	< 0.001 (< 0.001, > 999.999)	3.08 (0.35, 26.78)	7.65 (0.41, 141.78)	
Another/Multiple			< 0.001 (< 0.001, > 999.999)	< 0.001 (< 0.001, > 999.999)	< 0.001 (< 0.001, > 999.999)	3.41 (0.19, 59.69)	> 999.999 (< 0.001, > 999.999)	
Ever injected drugs			0.05 (0.00, 2.50)	1.31 (0.11, 15.39)	0.41 (0.04, 4.79)	0.48 (0.06, 3.82)	1.89 (0.07, 55.18)	
Sexual orientation Gay			1.00 (--)	1.00 (--)	1.00 (--)	1.00 (--)	1.00 (--)	
Bisexual			0.24 (0.03, 1.99)	0.34 (0.04, 3.03)	1.42 (0.26, 7.73)	0.32 (0.06, 1.57)	0.17 (0.02, 1.64)	
Heterosexual			-	> 999.999 (< 0.001, > 999.999)	> 999.999 (< 0.001, > 999.999)	> 999.999 (< 0.001, > 999.999)	1.15 (< 0.001, > 999.999)	
Homeless in past year			0.59 (0.07, 4,98)	0.74 (0.09, 6.13)	0.52 (0.08, 3.14)	1.38 (0.15, 13.03)	0.09 (0.01, 1.47)	
Venue type		Bar/Club	1.00 (--)	1.00 (--)	1.00 (--)	1.00 (--)	1.00 (--)	
Sex Environment/Street Location/Park			1.10 (0.09, 12.96)	< 0.001 (< 0.001, > 999.999)	0.60 (0.06, 6.52)	0.30 (0.04, 2.12)	0.56 (0.04, 8.66)	
Other			1.05 (0.00, 611.76)	1.72 (0.33, 8.91)	2.49 (0.27, 22.61)	1.11 (0.24, 5.09)	0.51 (0.03, 7.81)	

Models include all listed variables. Bold indicates statistical significance.

N represents the total number of observations in each wave. n represents the number of observations used in the estimation (no missing values).

MSM over 30 had lower odds of HIV testing in the past year compared to younger men from 2008 to 2014, but by 2017 there was no difference by age. In 2017, white MSM and bisexual or heterosexual MSM were less likely to report past year HIV testing, but these associations were not significant in later waves and no other variables were significantly associated.

### Average Predicted Probability of HIV Outcomes Over Time

**Fig. 1:**
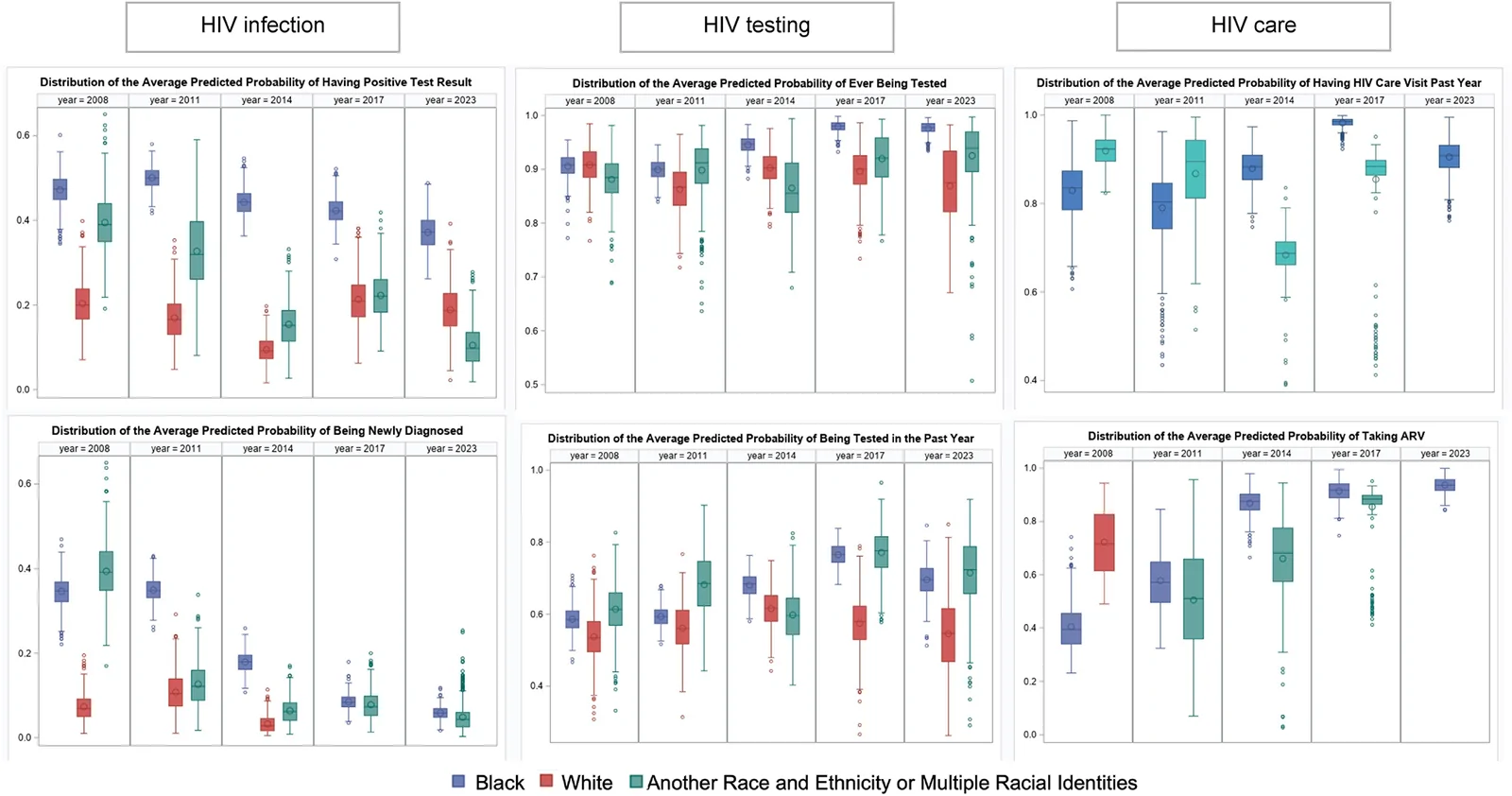
Average predicted probability of HIV infection and service utilization among gay, bisexual, same gender loving, and other men who have sex with men in Baltimore NHBS by race/ethnicity and wave, 2008–2023. Estimates for some racial/ethnic groups are suppressed for certain outcomes because the number of respondents in those categories was too small to report

Accounting for the demographic characteristics of the samples at each time point (Fig. 1), the average predicted probability (APP) of an HIV positive diagnosis decreased from 41% (CI: 35%, 46%) to 29% (CI: 23%, 35%) and the APP of unrecognized HIV infection decreased from 72% (CI: 62%, 81%) to 10% (CI: 6%, 16%). The APP for all other outcomes improved steadily from 2008 to 2023, with only a small decline in the APP of recent HIV testing and HIV care visit between 2017 and 2023. By 2023, the APP of ever testing neared 100% (APP 97%, CI 93%, 99%) and past year testing was close to 70% (APP 68%, CI: 61%, 75%). The APP of past year HIV care, ARV use, and viral suppression all exceeded 90%. Results of multiple pairwise comparisons show that shifts are statistically significant, with the exception that there is minimal change in the APP of ever HIV testing between 2017 and 2023 and ARV use between 2014 and 2017.

Stratified analyses show notable differences between Black and white MSM (Supplemental Table 1). While all outcomes improve among Black MSM, the APP changes in HIV prevalence and HIV testing remain stable among white MSM. As of 2023, APP of HIV infection among Black MSM is approximately twice that of white MSM (37% compared to 19%). APP of HIV testing exceeded 90% for both groups as of 2014 but diverged by 2023 with an increase to 98% (CI: 95%, 99%) among Black MSM and reduction to 87% (CI: 71%, 96%) among white MSM. APP of recent HIV testing was 70% (CI: 61%, 79%) among Black MSM and 54% (CI: 34%, 73%) among white MSM in 2023, both lower than the previous wave. APP of HIV care and ART use improved substantially to more than 90% in 2023.

## Discussion

This study summarized trends in HIV prevalence, testing, and care outcomes among venue-recruited MSM in Baltimore between 2008 and 2023. HIV prevalence remained relatively stable at approximately 2 of every 5 MSM throughout the study period. However, a closer look reveals enduring and stark racial disparities that continue to demand attention. The average predicted probability of testing HIV positive was almost three times higher among Black MSM compared to white MSM. In 2023, HIV prevalence was 44% among Black MSM compared to 16% among white MSM, ranking among the NHBS jurisdictions with the highest overall prevalence [15].

By 2023, almost all MSM reported having ever received an HIV test and approximately three-quarters of HIV-negative MSM had been tested in the past year. The proportion of MSM with HIV who reported a previous HIV diagnosis who were receiving care, taking antiretroviral drugs, and virally suppressed each reached the 90% threshold by 2017. Notably, the greatest improvements occurred among Black MSM. For this group, the likelihood of having seen a provider for HIV care and taking antiretroviral drugs doubled to almost 100% and almost all were virally suppressed by 2023. In contrast, past year care visits declined from 100% to 88% among White MSM diagnosed with HIV. Although the absolute numbers remained very low, it will be important to continue to monitor care engagement in this group.

Racial disparities have been a recognized central dimension of the HIV epidemic among Baltimore MSM since the earliest HIV behavioral surveillance studies in the early 2000s. The Young Men’s Study (YMS) found that HIV infection among 15–22 year old MSM was approximately 10 times higher among Black compared to white participants (27% compared to 3%) [20, 21], with similarly profound disparities in HIV incidence [22]. The first NHBS cycle in 2004 showed that the finding was not limited to young men. Using the same methods as YMS, Baltimore reported an overall HIV prevalence of 38% among adult MSM in 2004: 51% among Black MSM and 13% among non-Hispanic white MSM [13]. Further, 60% of Black MSM who tested HIV positive reported no previous HIV diagnosis compared to only 15% of white MSM.

The high HIV prevalence and glaring disparities revealed through behavioral surveillance were initially met by skepticism by public health authorities, but resonated among community members familiar with the underlying pervasive structural barriers such as poverty, racism, and stigma who quickly mobilized crisis response [23, 14, 24]. The numbers were essentially replicated in the next wave of NHBS (2008, reported herein) and followed by a concurrent spike in new HIV diagnoses among Black MSM in Baltimore HIV case surveillance and persistent racial disparities ever since [12].

In this context, the substantial decline in new HIV diagnoses and increased HIV service utilization between 2008 and 2023 -- especially the magnitude of change among Black MSM -- are particularly positive developments. These improvements likely reflect the combined impact of enhancements in Baltimore’s HIV program and service infrastructure alongside community advocacy and mobilization that transformed the surrounding social context, increasing knowledge of HIV status and willingness to disclose positive HIV status [25], both of which can facilitate improved HIV clinical outcomes [26].

From a public health and social justice perspective, these positive trends are very welcome despite their underlying roots. Over the past two decades, Baltimore City has invested heavily in the HIV response to address racial disparities and curb the epidemic among Black MSM. These have included workgroups and mobilization efforts, social marketing campaigns, multi-level interventions, expanded prevention and treatment services, and a diverse scope of applied research (including behavioral surveillance) to inform community needs and strengths – as well as the energy and commitment of a broad range of community members, advocates, researchers, providers, community-based organizations, health centers and health systems, with strong support and coordination from the Baltimore City and Maryland health departments.

Baltimore’s approach integrated evidence-based strategies and epidemiological modeling [27, 28] with a data-informed and community-oriented strategy that recognized the social and structural challenges underlying HIV and racial disparities among MSM [14]. Innovative targeted HIV testing initiatives engaged community members through mobile outreach and online platforms [29], combined syphilis and HIV services [30], collaborative house ball events [31], and venue-oriented strategies [32]. Efficacious behavioral [33] and biomedical interventions [34] based on social network theory emphasized peer support, stigma reduction, and supportive services. PrEP awareness and use increased with a patient-centered care approach [35] enhanced by provider feedback systems and rapid scale-up [36]. The trends reported here signal the success of these combined efforts. Yet, additional tailored responses are needed, evidenced by sexual orientation disparities within these data and the continued high prevalence of social and structural challenges affecting this population [37].

There are important limitations to consider. Data collection for NHBS is limited to MSM recruited from venues, which is not a full representation of all MSM in Baltimore. Except for HIV test results, all data are based on self-report and may be influenced by social desirability. Self-reported HIV infection may be subject to misreporting due to stigma or fear of study exclusion [25], but the positive trends are consistent across outcomes. The data are not weighted to account for potential venue selection bias or variability across venues beyond type, all of which may also have changed over time. The sample size was also lower in 2023 compared to prior waves, primarily due to fewer viable recruitment venues and available venue-time-location windows during the fixed recruitment period, which may affect statistical power and generalizability of the findings. Nevertheless, the 2023 sample maintained a similar demographic profile to earlier waves, and our findings are consistent with Baltimore HIV case surveillance data from the same year showing lower new HIV diagnoses among MSM and high rates of linkage to care and viral suppression [12]. Specifically, new HIV diagnoses among MSM were lower than in the earliest years of the epidemic, more than 90% of newly diagnosed individuals were linked to care within the first month and nearly half achieved viral suppression within six months. Finally, the data are all cross-sectional and associations should not be considered causal. Although self-reported prior participation was relatively low, it is possible that there is overlap in study participants over time that could inflate precision of estimates. Still, the best interpretation of the data are as serial point-in-time assessments of the population of MSM who attend MSM venues in Baltimore.

It is important to note that the relevance of venue-based recruitment for the diversity of the MSM population and the variety of ways that men choose to socialize has also changed over time. Venue-based time location sampling was initially selected for NHBS as a strategy well-suited for a social context in which partner seeking and community affiliation was largely centered around physical locations, i.e., bars, restaurants, street locations [16, 38]. Although it has always been recognized that VBS would not reach those MSM who do not attend venues where at least half of the attendees are likely to also be MSM, there was evidence that the VBS approach was likely to reach those at increased risk of HIV transmission. However, the social spaces where MSM congregate and find partners has changed dramatically during the study period. Internet sites and apps have largely supplanted physical venues for partner seeking [39]. Increased community acceptance of LGBTQ + populations has also expanded the options for safe public socialization. These factors have in turn contributed to a historic decline in the number of venues where LGBTQ + people concentrate in large numbers: many have closed or shifted to a more diverse clientele. These shifts contributed to slower recruitment and smaller sample sizes in recent NHBS cycles and may require methodological adaptation in future implementation.

This study reinforces both the necessity of health disparities research and the critical value of HIV behavioral surveillance activities. The legacy of the HIV epidemic reinforces the point made repeatedly by human rights scholars: infectious disease thrives at the intersection of marginalization and structural inequities [40]. This was starkly evident in the earliest days of HIV in the U.S. where heterosexism was a primary driver of delayed government response and inadequate funding that resulted in countless preventable losses. Yet even as this early discrimination was overcome to achieve incredible progress against the disease and rates of new HIV infections dropped among the broader population of MSM, the public health community missed the rise of infections among Black and African-American MSM [41, 42]. This oversight has been retrospectively attributed to a combination of cultural blind spots and explicit racism in HIV response.

Baltimore is now clearly on track to achieve ‘ending the HIV epidemic’ goals [43]. New diagnoses have declined steadily to a total of 166 in 2023, down from 1,027 in 2008 [12]. While MSM continue to account for the majority of new diagnoses, and Black MSM still bear a disproportionate burden, the progress achieved in Baltimore over the past 20 years is remarkable. However, the underlying social determinants of HIV remain entrenched [15], backlash against LGBTQ + policy gains is intensifying, and public health services and structural supports face increasing threats in the current federal landscape. As we celebrate Baltimore’s hard-won achievements, we should also recognize that we are at a critical juncture in the history of HIV response. Sustaining our progress demands unwavering commitment to equitable access to evidence-based HIV prevention and care services, robust funding for effective programs and resources, and continued investment in vital data systems such as NHBS that we know can tell us what we might otherwise miss.

## Supplementary Information

Below is the link to the electronic supplementary material.

Supplementary Material 1
