# Supplementary material for: Racial Disparities in HIV Prevalence, Testing, and Care Among Men Who Have Sex with Men in Baltimore from 2008 to 2023: Fifteen Years of HIV Behavioral Surveillance
Source: AIDS Behav. 2026 Feb 19;30(9):2646–57. doi: 10.1007/s10461-026-05031-7 (PMC13202490; doi:10.1007/s10461-026-05031-7)
Supplement: Supplementary file 1 [file 10461_2026_5031_MOESM1_ESM.docx]

Supplemental Table 1. Predicted probability of HIV infection and service utilization among Baltimore NHBS-MSM participants by race/ethnicity and wave, 2008-2023

|  | **Average Predicted Probability**  **(95% CI)** | | | | |
| --- | --- | --- | --- | --- | --- |
|  | **2008** | **2011** | **2014** | **2017** | **2023** |
| **HIV positive test result** | | | | | |
| Total | 0.41 (0.35, 0.46) | 0.42 (0.37, 0.47) | 0.34 (0.30, 0.39) | 0.35 (0.31, 0.40) | 0.29 (0.23, 0.35) |
| Black | 0.48 (0.39, 0.54) | 0.50 (0.45, 0.56) | 0.44 (0.38, 0.50) | 0.42 (0.36, 0.49) | 0.37 (0.29, 0.45) |
| White | 0.20 (0.11, 0.32) | 0.16 (0.08, 0.29) | 0.09 (0.04, 0.16) | 0.18 (0.10, 0.28) | 0.19 (0.08, 0.30) |
| Another or Multiple Race | 0.41 (0.26, 0.54) | 0.31 (0.16, 0.50) | 0.15 (0.07, 0.27) | 0.21 (0.12, 0.34) | 0.11 (0.04, 0.22) |
| **Newly diagnosed with HIV** | | | | | |
| Total | 0.30 (0.25, 0.34) | 0.28 (0.23, 0.33) | 0.14 (0.10, 0.18) | 0.07 (0.04, 0.09 | 0.04 (0.02, 0.06) |
| Black | 0.35 (0.27, 0.43) | 0.35 (0.30, 0.41) | 0.18 (0.13, 0.23) | 0.09 (0.05, 0.12) | 0.06 (0.03, 0.09) |
| White | 0.07 (0.03, 0.15) | 0.09 (0.03, 0.20) | 0.02 (0.01, 0.07) | - | - |
| Another or Multiple Race | 0.41 (0.26, 0.54) | 0.12 (0.03, 0.23) | 0.06 (0.01, 0.12) | 0.07 (0.02, 0.16) | 0.04 (0.01, 0.13) |
| **Ever tested for HIV** | | | | | |
| Total | 0.91 (0.88, 0.94) | 0.90 (0.87, 0.93) | 0.93 (0.91, 0.96) | 0.96 (0.94, 0.98) | 0.97 (0.93, 0.99) |
| Black | 0.91 (0.85, 0.94) | 0.90 (0.86, 0.93) | 0.95 (0.91, 0.97) | 0.98 (0.96, 1.00) | 0.98 (0.95, 0.99) |
| White | 0.90 (0.84, 0.97) | 0.87 (0.77, 0.94) | 0.90 (0.84, 0.96) | 0.90 (0.81, 0.96) | 0.87 (0.71, 0.96) |
| Another or Multiple Race | 0.89 (0.79, 0.96) | 0.94 (0.76, 0.98) | 0.84 (0.75, 0.99) | 0.90 (0.83, 0.99) | 0.94 (0.77, 0.99) |
| **Tested in the past year** | | | | | |
| Total | 0.59 (0.53, 0.64) | 0.60 (0.55, 0.65) | 0.66 (0.61, 0.72) | 0.74 (0.68, 0.79) | 0.68 (0.61, 0.75) |
| Black | 0.58 (0.51, 0.66) | 0.59 (0.54, 0.65) | 0.69 (0.62, 0.75) | 0.77 (0.71, 0.82) | 0.70 (0.61, 0.79) |
| White | 0.53 (0.41, 0.66) | 0.55 (0.43, 0.68) | 0.61 (0.51, 0.71) | 0.60 (0.46, 0.72) | 0.54 (0.34, 0.73) |
| Another or Multiple Race | 0.62 (0.47, 0.76) | 0.69 (0.48, 0.85) | 0.56 (0.46, 0.74) | 0.78 (0.64, 0.88) | 0.73 (0.48, 0.88) |
| **HIV care visit in past year** | | | | | |
| Total | 0.87 (0.65, 0.95) | 0.79 (0.56, 0.91) | 0.89 (0.82, 0.96) | 0.98 (0.91, 1.00) | 0.91 (0.76, 0.98) |
| Black | 0.83 (0.67, 0.95) | 0.82 (0.54, 0.94) | 0.88 (0.80, 0.95) | 0.99 (0.95, 1.00) | 0.92 (0.81, 0.98) |
| White | - | - | - | - | - |
| Another or Multiple Race | 0.92 (0.84, 0.98) | 0.94 (0.66, 0.97) | 0.71 (0.59, 0.78) | 0.89 (0.47, 0.92) | - |
| **Taking ARV** | | | | | |
| Total | 0.43 (0.31, 0.61) | 0.68 (0.50, 0.83) | 0.88 (0.78, 0.95) | 0.87 (0.77, 0.96) | 0.93 (0.85, 0.99) |
| Black | 0.33 (0.26, 0.61) | 0.63 (0.42, 0.81) | 0.87 (0.77, 0.95) | 0.92 (0.83, 0.98) | 0.95 (0.88, 0.99) |
| White | 0.70 (0.51, 0.92) | - | - | - | - |
| Another or Multiple Race | - | 0.55 (0.10, 0.87) | 0.77 (0.33, 0.88) | 0.89 (0.47, 0.92) | - |
| **Undetectable viral load** | | | | | |
| Total | - | - | - | 0.88 (0.78, 0.96) | 0.91 (0.79, 1.00) |
| Black | - | - | - | 0.87 (0.78, 0.97) | 0.997 (0.95, 0.999) |
| White | - | - | - | - | - |
| Another or Multiple Race | - | - | - | - | - |
